# ASSESSMENT OF ALVEOLAR BONE PATTERN IN OBESE AND NON-OBESE WOMEN, BEFORE AND AFTER BARIATRIC SURGERY: A PROSPECTIVE COHORT STUDY

**DOI:** 10.1590/0102-672020190001e1501

**Published:** 2020-07-08

**Authors:** Jefry Alberto VARGAS, Rafaela Carolina Soares BONATO, Eliel Soares ORENHA, Silvia Helena de Carvalho SALES-PERES

**Affiliations:** 1 Pediatric Dentistry, Orthodontics and Public Health Department, Bauru School of Dentistry, University of São Paulo, Bauru, SP, Brazil

**Keywords:** Obesity, morbid, Jaw, Prostheses and implants, Obesidade mórbida, Mandíbula, Implante dentário Subperiósteo

## Abstract

**Background:**

Bariatric surgery may have a negative impact on oral bone structure.

**Aim:**

To verify the alveolar bone pattern through radiomorphometric indices of panoramic radiography and linear measurements performed in periapical radiographs in eutrophic and morbidly obese patients before and after bariatric surgery.

**Methods:**

The sample consisted of 31 women aged 20-35 years old, divided into two groups: obese group (GO-obese grade III) and control group (GC-eutrophic). Twenty eutrophic and 11 obese morbidities were evaluated in the pre and postoperative bariatric surgery (six months). Radiomorphometric and plaque indices were evaluated at T0 (baseline) and T1 (six months) times, in both groups. In the radiographic analysis the trabecular pattern through the Lindh visual ladder and the bone loss were evaluated by calculating the distance from the cement-enamel junction to the bone crest in periapical radiographs. Panoramic radiographs were used to measure the mandibular cortical index (ICM), mentonian index (IM) and panoramic mandibular index (MPI), in addition to the Turesky plate index.

**Results:**

There was a significant loss of bone in T1 in patients submitted to bariatric surgery, when compared to eutrophic patients (p<0.05). The trabecular pattern became sparser after surgery with a visual difference. The plate index showed a slight improvement after surgery and the eutrophic maintained similar values over time.

**Conclusion:**

The standard alveolar bone presents greater bone loss in obese patients and worsens this standard after bariatric surgery when compared to eutrophic patients. The same happens with the trabecular pattern that becomes sparser after bariatric surgery.

## Graphical Abstract

[Fig f1a]

**Figure f1a:**
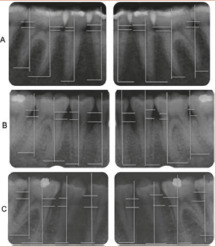
Right and left periapical radiographs in A eutrophic example of normal bone level; in B of morbidly obese before bariatric surgery, presenting bone loss; and in morbidly obese C after six months of the operation presenting great bone loss

## RESUMO

**Racional:**

A cirurgia bariátrica pode ter um impacto negativo na estrutura óssea bucal.

**Objetivo:**

Verificar o padrão ósseo alveolar por meio de índices radiomorfométricos da radiografia panorâmica e medidas lineares realizadas em radiografias periapicais, em pacientes eutróficas e obesas mórbidas antes e após a cirurgia bariátrica.

**Métodos:**

A amostra foi constituída por 31 mulheres com idade de 20 a 35 anos, sendo divididas em dois grupos: grupo obeso (GO-obesas de grau III) e grupo controle (GC-eutróficas). Foram avaliadas 20 eutróficas e 11 obesas mórbidas no pré e pós-operatório de operação bariátrica (seis meses). Índices radiomorfométricos e de placa dentária em ambos os grupos foram avaliados nos tempos T0 (baseline) e T1 (seis meses). Na análise radiográfica foram avaliados o padrão trabecular através da escada visual de Lindh e a perda óssea por meio do cálculo da distância da junção cemento-esmalte à crista óssea, em radiografias periapicais. Já as radiografias panorâmicas mensuram índice da cortical mandibular (ICM), índice mentoniano (IM) e índice panorâmico mandibular (IPM), além do índice de placa de Turesky.

**Resultados:**

Houve perda óssea significativa em T1 em pacientes do GO, quando comparada com as eutróficas (p<0,05). O padrão trabecular tornou-se mais esparso após a operação apresentando diferença visual. No índice de placa foi observada ligeira melhora após a operação e os eutróficos mantiveram valores similares ao longo do tempo.

**Conclusão:**

Pacientes obesas apresentam maior perda óssea, a qual piora após a operação bariátrica, quando comparada com pacientes eutróficas. O mesmo acontece com o trabeculado ósseo que se torna mais esparso após a operação.

## Graphical Abstract

[Fig f1as]

**Figure f1as:**
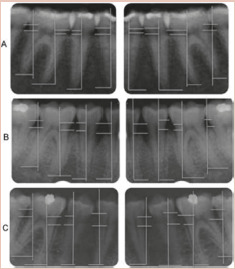
Radiografias periapicais direita e esquerda com exemplo em A de eutrófica com nível ósseo normal; em B de obesa mórbida antes da operação bariátrica, apresentando perda óssea; e em C de obesa mórbida após seis meses da operação apresentando grande perda óssea.

## INTRODUCTION

Obesity and overweight are defined as the accumulation of abnormal or excessive fat that can be detrimental to health. A simple way to measure obesity is body mass index (BMI), this is the weight of a person in pounds divided by the square of height in meters. A person with a BMI of 30 or above is considered obese and a BMI of 25 or above is overweight. Overweight and obesity are risk factors for many chronic diseases including diabetes, cardiovascular disease and cancer are included. Once considered problems in high-income countries, obesity and overweight are increasing in low- and middle-income countries, especially in urban areas^27^.

This disease is of multifactorial origin. The causes of obesity are manifold and include genetic, behavioral, nervous, endocrine and metabolic factors, as well as the type or lifestyle the individual adopts. Among the factors causing obesity, 30% can be attributed to genetic factors, 40% non-hereditary and 30% to the purely social, that is, the relationship between genetic and environmental factors is 30% and 70% respectively^16^.

As one of the main clinical treatments and as one of the most effective solutions, there is bariatric surgery. It promotes a significant reduction in excess body weight^2^, inducing a mean loss of 60% to 75% of excess body weight, with a maximum weight loss between 18-24 months postoperatively^3^. There are several oral health studies involving obesity^1^ ^,^ ^8^ ^,^ ^16^ ^,^ ^28^ ^,^ ^29^ and bariatric surgery^17^ ^,^ ^18^.

In bariatric surgery, active calcium absorption, 80% of which occurs in the duodenum and jejunum, is harmed^19^. There is clear evidence that in both, adolescents and young adults, despite increased mechanical load and regardless of lean mass, adipose tissue is not beneficial for bone structure^10^.

In addition, vitamin D absorption that occurs in the jejunum and ileum is affected by mixing ingested nutrients, which is retarded with bile acids and pancreatic enzymes. Secondary hyperparathyroidism and bone loss may develop as a consequence^10^.

Another challenge is microbial, since the increased prevalence of periodontal disease in obese adults may be associated with the high frequency of food consumption and, consequently, the increased accumulation of plaque, which may be aggravated due to poor oral hygiene^28^. Recent studies have shown a high percentage of gingival bleeding, periodontal pocket and insertion loss in obese individuals when compared to those of normal weight and the association between obese patients and alveolar bone loss from leptin alteration has already been demonstrated^4^.

The influence of general health problems on the osseointegration process as such is poorly documented^10^. So far, the question of what happens in the pre and postoperative jaws of patients undergoing bariatric surgery has not been very clear, as well as what would happen if one of these patients received dental implants as rehabilitative treatment.

Thus, this study aims to elucidate the scientific knowledge about bone pattern and alveolar bone resorption in morbidly obese patients before and after bariatric surgery and to relate them to people with normal weight.

## METHODS

This research was designed to meet the guidelines of the Declaration of Helsinki (1964), and was sent and approved by the Ethics Committee on Research with Human Beings, Bauru School of Dentistry, University of São Paulo (FOB/USP) and Amaral Carvalho Hospital of Jaú, was approved by the opinion CAAE: 45794415.9.0000.5417.

### Study type and configuration

STROBE^24^ guidelines were used to report this prospective cohort study conducted at the Bauru School of Dentistry at the University of São Paulo and was conducted from July 2015 to October 2016.

### Sample composition

The participants were recruited from the analysis of the medical records of the following clinics: Clinic of Patients with Eating Disorders and Obesity, Implantology, Periodontics, Integrated Rehabilitation, Bauru School of Dentistry, University of São Paulo, Bauru, SP, Brazil. Patients from the Amaral Carvalho Hospital were treated for treatments that restore or preserve the oral health of these patients before and after undergoing bariatric surgery at the institution previously mentioned.

To compose the obese group (obese grade III, BMI>40 kg/m^2^), patients who are normally attended at the Graduate Clinic, Collective Health Area, Disciplines I and II of Applied Clinical Practice Eating Disorders and Obesity were voluntarily invited to participate. To compose the control group - GC (eutrophic, BMI 18.5 to 24.99 kg/m²), the patients of the Screening of the School of Dentistry of Bauru were duly authorized by the responsible. If it was necessary to recruit patients from other oral health care facilities, the corresponding authorizations were previously requested and sent to the ethics committee. Similarly, for this group it was also not necessary to expose these patients to any additional treatment, that is, the treatment plan proposed by the professional/graduate student was not changed, not even the panoramic and periapical radiographs, since only those patients who were needed such radiographs.

It was not necessary to expose them to any additional treatment, that was, the treatment plan proposed by the professional/postgraduate student was not altered, not even the panoramic and periapical radiographs, only those patients in whom the radiographs were performed, were selected, in reason for treatment and not because of the present research. The experimental group (obese) and the control group (eutrophic) were evaluated at two times (T0=before surgery; T1=six months after surgery). The patients were only clinically evaluated for the Turesky plaque index where they chewed an Eviplac^®^ tablet to visually assess the lingual and buccal faces. Finally, periapical and panoramic radiographs were evaluated from the radiomorphometric indices, namely: mandibular cortical index (MCI), mental index (MI), panoramic mandibular index (MIP), bone level loss and trabecular pattern evaluation.

### Eligibility criteria

Patients with complete permanent dentition up to the left and right lower first molars were included, and those who underwent good quality panoramic and periapical radiographs of the lower premolar region showing a distinct trabecular pattern. Radiographic shots were performed before orthodontic treatment began or two years after orthodontic treatment.

Patients using glucocorticoids, anticonvulsants, antidepressants, bisphosphonates and immunosuppressants were excluded and also those diagnosed with osteoporosis or diabetes, the smokers, pregnant or nursing women, those who did not receive panoramic and periapical radiographs in the service, hypertensive women, and those with previous diagnosis of periodontal disease.

### Study design

The study consisted of the following steps: a) examiner calibration; b) radiographic examination by panoramic and periapical radiographs at T0 and T1. Twenty eutrophic patients with a BMI of 18.5 to 24.99 kg/m^2^ and a mean age of 28.5 years who composed the control group (GC) and 11 grade III obese patients with a mean age of 30 years, with a BMI>40 kg/m^2^ were evaluated composing the obese group (GO) and in the age group of 20 to 35 years. Both groups were evaluated at two times: (T0) before and (T1) after six months. GO was submitted to bariatric surgery.

### Image evaluations

#### Panoramic radiographs (radiomorphometric indices)

In the radiographic study of the eutrophic and obese, panoramic and periapical radiographs indicated for teeth extraction, bone loss, third molar extraction and areas of rehabilitation with dental implants were used. Radiographs that were not of good quality were excluded from the sample, considering the patient’s positioning, density, contrast and details, according to previously established norms.

The linear measurements were performed at T0 and T1, using a specialized analytical software, which offers 30% magnification correction, to better simulate the clinical situation. The radiographs were measured to obtain the radiomorphometric indices mentioned above, considering that: 1) mandibular cortical index (ICM) referred to the lower mandibular cortical height, assessing its degree of resorption and was classified into three groups^7^ being C1 normal (cortical margin would be clear and sharp on both sides), C2 osteopenic (the endosteal surface showed semilunar defects, lacunar resorption or cortical residues), C3 osteoporotic (very porous cortical layer); 2) mentonian index (IM), which measured the thickness of the mandibular cortex in the region of the mental foramen and identified and the line drawn perpendicular to the tangent of the lower border of the mandible and through the center of the mentonian foramen, with the cortical thickness measured at this point (normal value: greater than or equal to 3.1 mm)^11^ ^,^ ^13^ ^,^ ^22^; 3) mandibular panoramic index (MPI) calculated as the ratio of the lower mandibular cortical thickness in the mentonian region to the distance between the lower mandible edge and the lower margin of the mentonian foramen (normal value: greater than or equal to 0.3).

#### Periapical radiographs - radiographic standardization

The film used was Kodak Insight, the most sensitive film. The technique of parallelism was used at times T0 and T1, with the aid of the use of radiographic positioners. Exposure time was 0.14 s by radiography using 65 Kv apparatus. Exposure time and film used were the same for all patients. The films with the images were stored in an individual case free of moisture and light. Radiographic processing was manual. The developer and fixer used were the same for all radiographs, being new. Thermometer was used to check the developer temperature, leaving the film the time required according to the temperature indicated by the manufacturer. The steps were^6^: 1) film placed on the developer making movements for 5 s so as not to incorporate bubbles and then left unmoving during development; 2) after the set time, the film was removed from the developer by letting excess flow into the intermediate wash tank, and placed in water and stirred continuously in vertical movements for 20 s; 3) excess water was drained and then the film was placed in the fixative for 5 min; 4) excess fixative was drained into the wash intermediate tank and left for final wash for 10 min in running water; 5) drying of the radiography.

### Measurement of the images

Radiographs were scanned on a slide scanner with 800 dpi resolution and 8 bits. The images were saved in bitmap format. It was used for linear measurement or computer program Image J. Long axis tooth tracking was performed, and then tracked as lines that passed through the enamel junction (JCE), bone level closest to the tooth and root apex. All these lines must be perpendicular to the line drawn on the long axis of the tooth. The distance from the JCE to the bone level (JCE-CO) and the distance from the JCE to the root level (JCE-AR) were measured by the following calculation: JCE-CO/JCE-AR x 100, distal from the first pre -molar (34 and 44), mesial and distal of the second premolar (35 and 45) and mesial of the first molar (36 and 46). When the value exceeded 10% was considered as bone loss.

#### Evaluation of trabecular pattern

At T0 and T1, the evaluated sites were: areas between the first molar and the second lower premolar and between the first and second lower premolars. For the assessment of trabecular pattern, a visual scale^14^ was used, considering the scores: 1 as sparse (intertrabecular grid spaces, especially in the cervical region of the premolars); 2, as dense and sparse (trabecular denser in the cervical and more sparse apically), and 3, as dense (the whole area has the same degree of trabecular and the intertrabecular spaces were small).

#### Plaque Index

The presence or absence of plaque was assessed using the Turesky plaque index^23^. Prior to the evaluations, the subjects were exposed to plaque through mouthwash with basic fuscin solution (Eviplac, Biodynamics, Brazil), dispensed in disposable plastic cups and according to the instructions provided by the manufacturer. The examinations were performed using a flat buccal mirror and wooden spatula. The buccal and lingual surfaces of each tooth were evaluated and then assigned scores from 0 to 5, as follows: 0 (no plaque); 1 (separate portions or discontinuous strip of plaque at the cervical margin of the dental surface); 2 (separate portions or continuous range up to 1 mm of plaque on the cervical surface margin); 3 (plate covering up to 1/3 of the surface); 4 (plate covering between 1/3 and 2/3 of the surface); 5 (plate covering 2/3 or more of the surface).

The bacterial plaque count per individual was obtained by summing all plaque scores divided by the number of faces examined, with a possible amplitude of 0 to 5.

After the evaluation, the subjects underwent professional prophylaxis using a rubber cup, prophylactic paste and dental floss, as well as receiving oral hygiene guidelines.

### Statistical analysis

The independent variables were: time, group and plaque index. The dependent variables for comparison between groups were: bone density loss (MCI), mental index (IM), panoramic mandibular index (IPM), bone level loss (PNO), and trabecular bone. The collected and annotated data were organized in Microsoft Excel 2011 files. The results were presented in a descriptive part, in the form of tables and graphs, in which the data (variables) were presented as minimum, maximum, mean and standard deviation. In the statistical analysis the data normality and homogeneity test was initially applied, and then the appropriate statistical test was used. Among the groups with favorable and unfavorable results, the continuous and categorical variables were compared using the ANOVA and chi-square test, respectively. Regarding the favorable results, the odds ratio for each variable was calculated using univariate and multivariate logistic regression analysis. First, univariate analysis was performed for all variables associated with unfavorable outcomes. Variables that were significantly associated with unfavorable results with p <0.2 in the univariate analysis were entered into the multivariate model, together with potentially important variables such as group and age, regardless of statistical significance.

## RESULTS

### Evaluation by images

#### Periapical radiographs

#### Bone loss

Periapical radiographs evaluated bone loss at distal teeth 34 and 44, mesial and distal teeth 35, 36, 45, 46.

**FIGURE 1:**
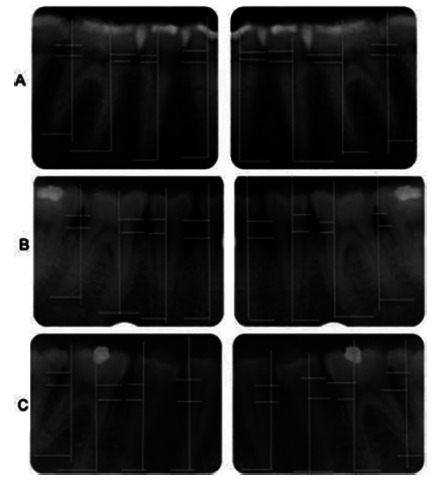
Right and left periapical radiographs in A eutrophic example of normal bone level; in B of morbidly obese before bariatric surgery, presenting bone loss; and in morbidly obese C after six months of the operation presenting great bone loss.

Figure 1 shows the periapical radiographs (right and left side) with the lines drawn in order to perform linear measurements to assess alveolar bone loss.

#### Evaluation of trabecular pattern

**FIGURE 2:**
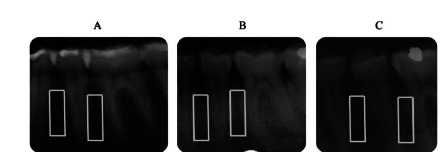
Areas of assessment of the trabecular pattern, having as an example, in A, score 3 (dense trabecular) in eutrophic; in B, score 2 (dense trabecular and sparse) in morbidly obese patient before the operation; and in C, score 1 (sparse trabecular) in a morbidly obese patient six months after the operation.

Periapical radiographs also assessed the trabecular pattern according to the scale of Lindh et al (1996)^15^. Figure 2 shows the demarcation of the evaluated regions.

**FIGURE 3:**
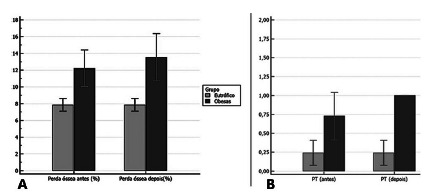
A) It can be observed the degree of bone loss where the GO in T0 had already presented it significantly, and in the greater T1 (1.8%) and the CG had no bone loss in T0 and T1; B) it can be observed in the evaluation of the trabecular pattern that the EG at T0 presented score 2 (dense and sparse trabecular) and at T1 score 1 (sparse trabecular), the latter worse, and the GC score 3 (dense trabecular) at T0 and T1.

The outcome of the bone loss assessment can be assessed in Figure 3.

Normal value=less than 10%; PT=trabecular pattern

#### Panoramicradiographs

#### Mandibular Cortical Index (ICM)

**FIGURE 4:**
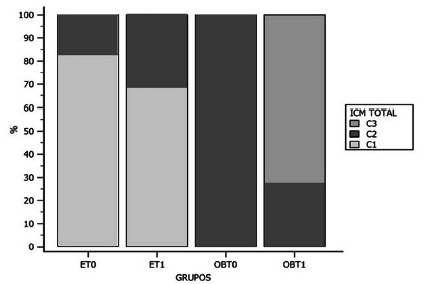
The degree of inferior mandibular cortical resorption can be observed (Klemetti et al., 1994)^11^ where the GO in T0 presented C2 (osteopenic) and in T1 presented C3 (osteoporotic), the latter being worse. The CG presented C1 (normal) at T0 and T1 evaluation.

In Figure 4, the panoramic radiograph beams evaluated the resorption of the mandibular cortex.

ET0=eutrophic patients (CG) before; ET1=eutrophic patients (CG) after six months; OBT0=morbidly obese (GO) patients before bariatric surgery; OBT1=morbidly obese patients (GO) after six months of operation

#### Mentonian Index (MI)

**FIGURE 5:**
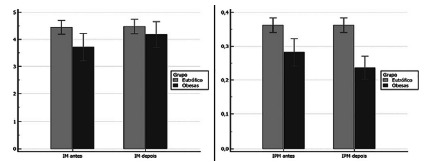
A) Result of evaluation of lower mandibular cortical thickness in the mental foramen region (normal value: greater than or equal to 3.1 mm); B) Mandibular Panoramic Index assessment result (normal value: greater than or equal to 0.3)

The panoramic radiographs also assessed the thickness of the lower mandibular cortex in the region of the mental foramen as shown in Figures 5A and 5B.

IM=mandibular Index; IPM=panoramic mandibular index

It can be observed in Figure 5A the lower mandibular cortical thickness in the region of the mental foramen (normal value: greater than or equal to 3.1 mm) where the GO in T0 were within normal values but presented a value lower than the GC. At T1, the EG showed an improvement in the mandibular cortical thickness, but the CG did not change at T0 and T1.

#### Mandibular panoramic index (IPM)

It can be observed in Figure 5B the panoramic mandibular index (MPI) (normal value: greater than or equal to 0.3) where the GO in T0 presented an altered average value (0.27) and presenting worse in T1 (0, 23). The CG was within normal limits at T0 and T1 (0.36).

#### Plaque Index

**FIGURE 6:**
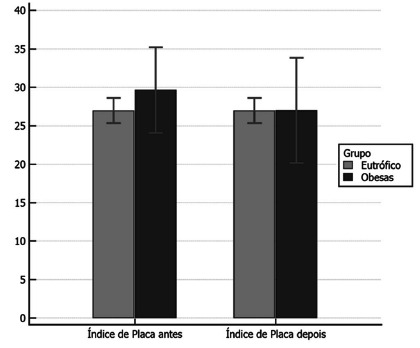
Turesky’s plaque index can be observed, where the GO in T0 presented an average value, 0 higher than in T1. The CG presented the same values at T0 and T1.

Turesky’s plaque index^23^ was clinically evaluated as shown in Figure 6.

## DISCUSSION

The results of the present study allow us to state that after bariatric surgery, the patients investigated showed increased alveolar bone loss and the trabecular bone was even more sparse, factors that are relevant for clinical application, since they may present tooth loss and seek for implant treatment, and may have increased tooth loss and resorption of the mandibular cortex, compromising masticatory function.

In the present study, the sample consisted of women aged between 20 and 35 years (± 29), being considered young. This population requires attention as obesity rates worldwide are increasing^6^. Obesity tends to persist throughout life.

The two constituent parts of the alveolar bone, dense cortical bone (external) and trabecular bone (internal), as well as the alveolar bone level (bone loss) were evaluated. Before the surgery (T0) the group of morbidly obese (GO) presented bone loss in 12.4%, which after six months (T1) was worse in the evaluated sites (13.6%, Figure 3). When comparing T0 and T1 independently of the group, there was no significant change (8%). However, there was a significant loss in GO when compared to CG, a fact that can have serious consequences in oral rehabilitation treatments, such as dental implants.

In a previous study conducted with premenopausal women to verify bone mineral density, it was shown that obese women had a lower proportion of dense bone trabeculae than non-obese women^20^.

There is still no scientific evidence regarding bone loss and its action in the osseointegration process of implants in morbidly obese pre and postoperatively. It takes about four months to complete^3^ and if followed by the operation will favor further bone loss impairing the bone repair process of the implant.

From the periapical radiographs, an increase in bone loss can be observed in GO patients after bariatric surgery. This loss directly impacts the dental treatment to be offered both, preoperatively and postoperatively which, depending on the case, may lead to implant loss.

The trabecular bone was evaluated in its external portion followed by the internal evaluation. According to White (2002)^25^ it is susceptible to physiological remodeling throughout life and may be influenced by masticatory function, orthodontic movements and tooth extractions. Thus, the present study excluded patients who used orthodontic appliances or had removed them less than two years ago, in addition to those who had extracted teeth in the area to be evaluated or in the adjacent region.

The trabecular pattern found in obese women favors faster bone loss due to its low density and occurs especially in women with osteoporosis^20^ ^,^ ^25^. This can get worse after surgery, as shown by the results of this research. Our results allow us to state that there is a risk factor for future dental implant installation in morbid and operated obese patients.

In physiological remodeling, resorption and formation are coupled and dependent phenomena, and the predominance of one over the other can result in gain or loss of bone mass^9^. Thus, in the present study, the mentonian index (IM) was evaluated, where it was observed that the GO in T0 presented normal values ​​(3.6 mm) but lower than the CG (4.4 mm). OG showed improvement in mandibular cortical thickness at T1 (4.3 mm), but the CG did not differ between T0 and T1. Future studies are necessary to verify the behavior in the T0 and T1 of GO, because significant changes could be presented in the long term. These results are in agreement with those of Lindh et al., (2004)^15^ in which there has been a positive correlation between bone mineral density and fat mass, suggesting that they are related to each other, which could be contributing with this difference in mandibular cortical thickness at T0 and T1 of GO. These results show the care that should be taken in patients with atrophic jaw who will be rehabilitated with dental implants to avoid fractures by the chewing forces exerted on the implant-supported prosthesis.

A new molecular link between obesity, chronic inflammation and periodontal disease has been investigated: leptin. It is an adipokine that may have an influence on bone metabolism. Sales-Peres et al. (2019)^20^ showed alveolar bone loss in obese patients, a factor that may compromise oral rehabilitative treatment. An action of leptin on bone metabolism is still controversial as it is involved in less than two different bone control mechanisms, directly stimulating growth or indirectly suppressing bone formation. To investigate a possible correlation between bone loss and bone metabolism was adopted or the non-GO panoramic mandibular panoramic index (IPM) during T0, the altered mean value (0.27) worsened at T1 (0.23) without significant difference (p>0.05). In the CG, the IPM was within the normal range at T0 and T1 (0.36), values ​​above 0.30. Future studies to verify the behavior of IPM in non-preoperative and postoperative patients are recommended, as they exhibited applicable changes, such as interfering with future rehabilitation treatment.

It is noteworthy that in the mandibular cortical index (MCI), all GO patients in T0 presented incipient porosity in the mandibular cortex, being classified according to Klemetti et al.,^11^ as osteopenia (C2), different from the group of eutrophic patients that, in their entirety in both T0 and T1, they presented normal index (C1).

The problem of osteopenia may be related to age, with bone tissue losing mass over the years. Bone mass loss begins around age 35 and continues at different rates throughout life^9^. Also according to Shapes et al.^21^ it is necessary to eliminate possible biases such as hormonal influences to perform specific analyzes. Thus, in the present study, the age range of the selected women was from 20 to 35 years old (premenopausal); therefore, both GO and CG did not present during menopause.

In the T1 GO, seven of the 11 patients had osteoporotic ICM (C3), with a very porous mandibular cortex representing the worst condition in this index^7^. According to WHO^26^, bone loss is directly related to age and is asymptomatic; osteoporosis is a determining factor in the occurrence of fractures, which in turn can lead to other morbidities, increasing mortality.

The presence of bacterial biofilm, also known as plaque, can trigger the two most prevalent oral diseases: dental caries and periodontal disease. The latter is initiated by the presence of bacterial biofilm and perpetuated with the dysregulation of the immune system in the gingival tissue. Among the explanations for the association between obesity and periodontal disease, we can mention the qualitative/quantitative presence of bacterial plaque^29^. Thus in the present study, patients with a previous diagnosis of gingivitis or periodontitis were excluded, thus eliminating possible biases that could interfere with the results.

Regarding the plaque index, which is a quantitative marker of supragingival bacteria, it was observed that GO in T0 presented the highest amount of plaque. However, comparing both groups, GO and GC, at T1, they showed that the GO group presented less plaque after six months of the operation. This demonstrated that the alteration in the alveolar bone level of the patients was not due to the amount of bacterial plaque and could be related to their quality. The quality of plaque, that is, the bacteria that were present in patients’ mouths, were not investigated in this study.

Bariatric surgery is considered the most effective tool to control and treat obesity and is directly related to periodontal disease^12^. Studies on clinical insertion level, probing depth and bacteria such as *Phorphyromonas gingivalis* in the postoperative period showed an increase in T1, increasing infectious foci in the mouth and decreasing quality of life^18^.

Evidence has shown that there is a relationship between obesity and bone loss in young adults^20^. Therefore, dental follow-up of morbidly obese patients should be performed in order to prevent the appearance of bone tissue complications, interfering with the quality of life of these patients. As shown in the present study, young obese women had greater bone loss both before and after six months, being worse in the latter period. These signs must be controlled early as they compromise rehabilitative treatments such as dental implants. These signs may directly interfere with patients chewing capacity^27^ ^,^ ^8^ and they will undergo hormonal changes in the future that directly interfere with bone remodeling (menopause and osteoporosis). These facts further aggravate bone loss after the operation^20^.

Thus, follow-up, treatment, patient monitoring and control of risk factors should be recommended to prevent postoperative complications^5^ and dental implant losses in future rehabilitation treatments.

## CONCLUSION

The alveolar bone pattern presents greater bone loss in obese patients and worsens after bariatric surgery when compared to eutrophic patients. The same is true of the trabecular pattern that becomes sparser postoperatively and may compromise osseointegration of dental implants in oral rehabilitation.
